# Construction of self-learning classroom history teaching mode based on human-computer interaction emotion recognition

**DOI:** 10.3389/fpsyg.2022.949556

**Published:** 2022-07-27

**Authors:** Changwei Ji, Shuyan Zhao

**Affiliations:** ^1^College of Culture and Tourism, Heihe University, Heihe, China; ^2^Academic Affairs Office, Heihe University, Heihe, China

**Keywords:** human-computer interaction, emotion recognition, autonomous learning, teaching mode, history class

## Abstract

Due to the continuous epidemic in recent years, the traditional teaching mode of history classroom has been gradually replaced by the teaching mode of self-learning classroom. The teaching mode of autonomous learning classroom has become a popular teaching mode in recent years. However, in the autonomous learning classroom under the current history teaching mode, the lecturer cannot always pay attention to the various states of the students. It is also difficult to understand and receive the information the teacher wants to convey in real time. For this reason, human-computer interaction emotion recognition technology has been proposed and developed. In order to construct and realize the teaching mode of self-learning classroom history, this paper studies the emotion recognition technology of human-computer interaction. The research results show that the introduction of human-computer interaction emotion recognition technology into the construction of autonomous learning classroom teaching mode can fully understand students' emotional behavior. It improves the accuracy of students' emotion recognition by 2.67%, enables students to maintain a good learning motivation, and make reasonable plans and arrangements for the historical time and progress of learning. At the same time, it enhances the history teaching intensity and autonomous learning ability, and improves the original single learning mode. By establishing a new teaching-teacher-student relationship, it creates a good and active autonomous classroom atmosphere.

## Introduction

The concept of autonomous learning classroom is the most concerned teaching mode concept since the new century, and it is also a basic feature of the new educational philosophy. However, because the self-learning classroom emphasizes the enthusiasm and initiative of students to receive knowledge, it still does not get rid of the shadow of teacher-centered theory (Chau et al., 2021). It regards students as the object of teaching rather than the main body of autonomous learning, which is easy to cause the deviation of students' learning of historical knowledge. Coupled with the inherent drawbacks of the traditional teaching model, it has led to a wealth of research on autonomous learning classrooms. However, the research on human-computer interaction emotion recognition is very lacking. Therefore, it is very important to combine human-computer interaction emotion recognition and autonomous learning classroom teaching mode.

Self-directed learning classrooms can be carried out in the form of individual self-study, cooperative learning, and inquiry learning. It attaches great importance to the connection with real life, and to a large extent can facilitate students to discuss and exchange. In order to promote the construction and development of history teaching classrooms, many people have conducted research on this. Zhang W employed a mixed methods design to investigate the impact of seven classroom assessment features on students' self-regulated learning, and further explored the influencing factors (Zhang, 2017). Since second language motivation can have a crucial impact on learners' learning outcomes, Chen (2022) took the second language motivational self-system as a theoretical framework to explore the influence of learners on their second language learning performance. Zheng et al. (2020) compared the impact of two mind mapping strategies combined with teaching methods on student learning outcomes. Because motivation and success in computer science courses are influenced by the strength of students' self-efficacy beliefs about their learning ability, students with weaker beliefs may have difficulty succeeding in computer science courses. Srisupawong et al. (2018) investigated the factors that enhance or hinder computer self-efficacy among computer science students. Dignath (2021) applied a generic model of teacher competence to the specific context in which teachers promote autonomous learning in the classroom, and investigated teacher competence profiles in terms of competence and how teacher competence modulates the effectiveness of self-regulated learning. As the COVID-19 crisis continues, the need to resume virtual learning opportunities is seriously felt (García et al., 2021). Khodaei et al. (2022) aimed to determine the impact of online flipped classroom on self-directed learning readiness and metacognitive awareness of nursing students. Since teachers play a key role in supporting children's metacognition and learning in the curriculum, Loon et al. (2021) aimed to gain insight into teachers' teaching with cognitive and metacognitive strategies, and the relationship between teacher-oriented and child-centered teaching practices and children's self-monitoring accuracy, learning regulation, and learning performance. From this point of view, the research results on the autonomous learning classroom are already very rich, but none of these research results have combined the autonomous learning classroom with the history teaching mode for research. In order to solve this problem, this paper studies the combination of the two.

There are more and more human-computer interaction systems in the teaching field, and it is an inevitable trend in the future to improve the service quality of human-computer interaction emotion recognition and make it more intelligent. In order to promote the development of human-computer interaction emotion recognition technology, many people have carried out research on it (Grange and Barki, 2020). In order to effectively optimize the accuracy of emotion recognition, Chen et al. (2021) proposed a novel emotion recognition framework based on machine learning. The core of the framework is to select the optimal classifier for different emotional data, fuse the classification results of each classifier, and obtain the global classification result. To enable machines to recognize human emotional states and respond intelligently to human needs, Jaratrotkamjorn (2021) proposed an emotion recognition system through a multimodal approach that integrates information from facial and speech expressions. Since the domain difference between training data and test data is still a major challenge to achieve improved systems to achieve emotion recognition performance, Zheng introduced a new multi-scale difference adversarial network. It is designed to narrow the gap between the source and target domains (Zheng et al., 2021). Since the current facial recognition features are not well-defined, Kommineni et al. (2021) proposed a method that can improve high performance computing in terms of facial expression recognition accuracy. Because the increasing use of computing devices and applications in human daily life has triggered the need for natural human-computer interaction, Mukeshimana et al. (2020) proposed emotion recognition using multiple features using a semi-serial fusion method. Since speech emotion recognition has become the core of most human-computer interaction applications in the modern world, Zvarevashe and Olugbara (2020) developed a custom 2D convolutional neural network that can perform feature extraction and classification of sounds. In order to make human-machine interfaces more effective and develop human-like systems, Peerzade et al. (2018) elaborated the basics of speech emotion recognition systems and reviews different feature extraction and classification techniques for the systems.

Emotion recognition has important implications for teachers and students in autonomous learning classrooms using human-computer interaction systems. Some excellent human-computer interaction emotional technologies not only meet the learning needs of teachers and students, but also judge the emotional state of students according to the results of emotion recognition, so as to provide teachers with more intimate teaching suggestions and program services. Therefore, emotion recognition is very important for human-computer interaction and autonomous learning classrooms, but in the field of current teaching models, there are still big problems in the development and application of human-computer interaction emotion recognition technology and autonomous learning classrooms. In order to solve these problems, this paper studies the construction of history teaching mode in autonomous learning classroom based on human-computer interaction emotion recognition.

## Construction content of autonomous learning classroom history teaching mode

### The overall framework of the human-computer interaction emotion recognition system

**Figure 1:**
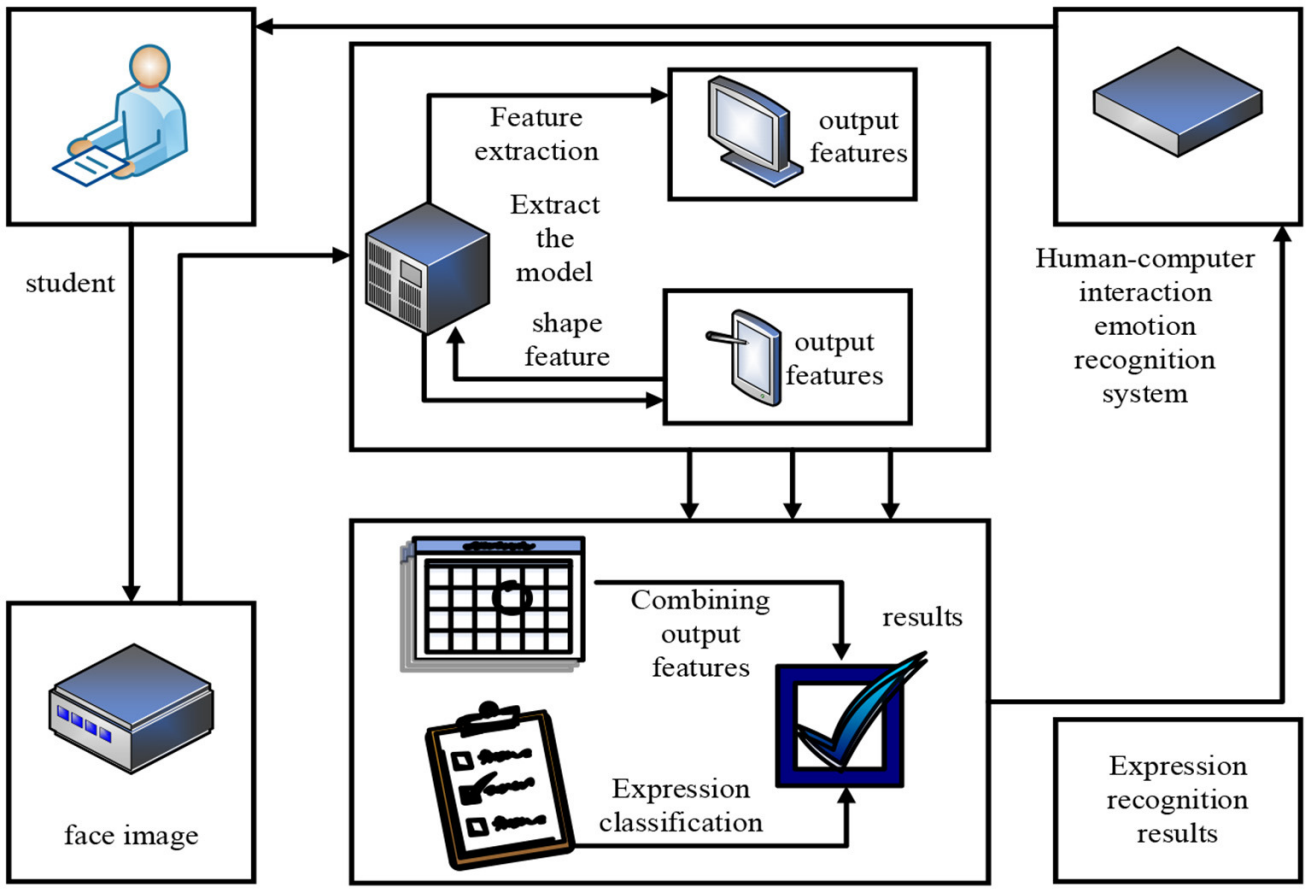
The overall framework of human-computer interaction emotion recognition.

The application of facial expression recognition in intelligent robots is mainly reflected in intelligent human-computer interaction systems (Bota et al., 2019). The intelligent human-computer interaction system based on expression recognition is mainly composed of face detection, expression feature extraction, expression classification and interaction strategy modules. In order to construct a teaching model for autonomous learning of classroom history, this paper studies the human-computer interaction emotion recognition system, and its overall framework is shown in Figure 1.

As can be seen from Figure 1, the main process of the human-computer interaction emotion recognition system is five steps: human-computer interaction, capturing face images, feature extraction, emotion recognition, and returning the recognition results. The specific process of human-computer interaction emotion recognition is as follows. When students study in the autonomous learning class of the history teaching mode, the intelligent machine will capture the student's face image through the camera. And it feeds it into the feature extraction module to identify students' states and emotions when they study history. In the feature extraction module, the intelligent machine will extract the texture and shape features of the captured face image through the face recognition model. And it eliminates various interference factors other than the face image, so as not to affect the later face image feature extraction and classification. After feature extraction and classification, the feature extraction module communicates its results to the expression recognition module in the form of geometric features of the shape model. In the expression recognition module, the combined output shape and texture features are used for expression recognition. And it classifies different emotions, and it is enough to get specific results for teachers' reference and continuous improvement of teaching methods, so as to increase students' interest in learning.

### Overall framework of human-computer intelligent interaction system

**Figure 2:**
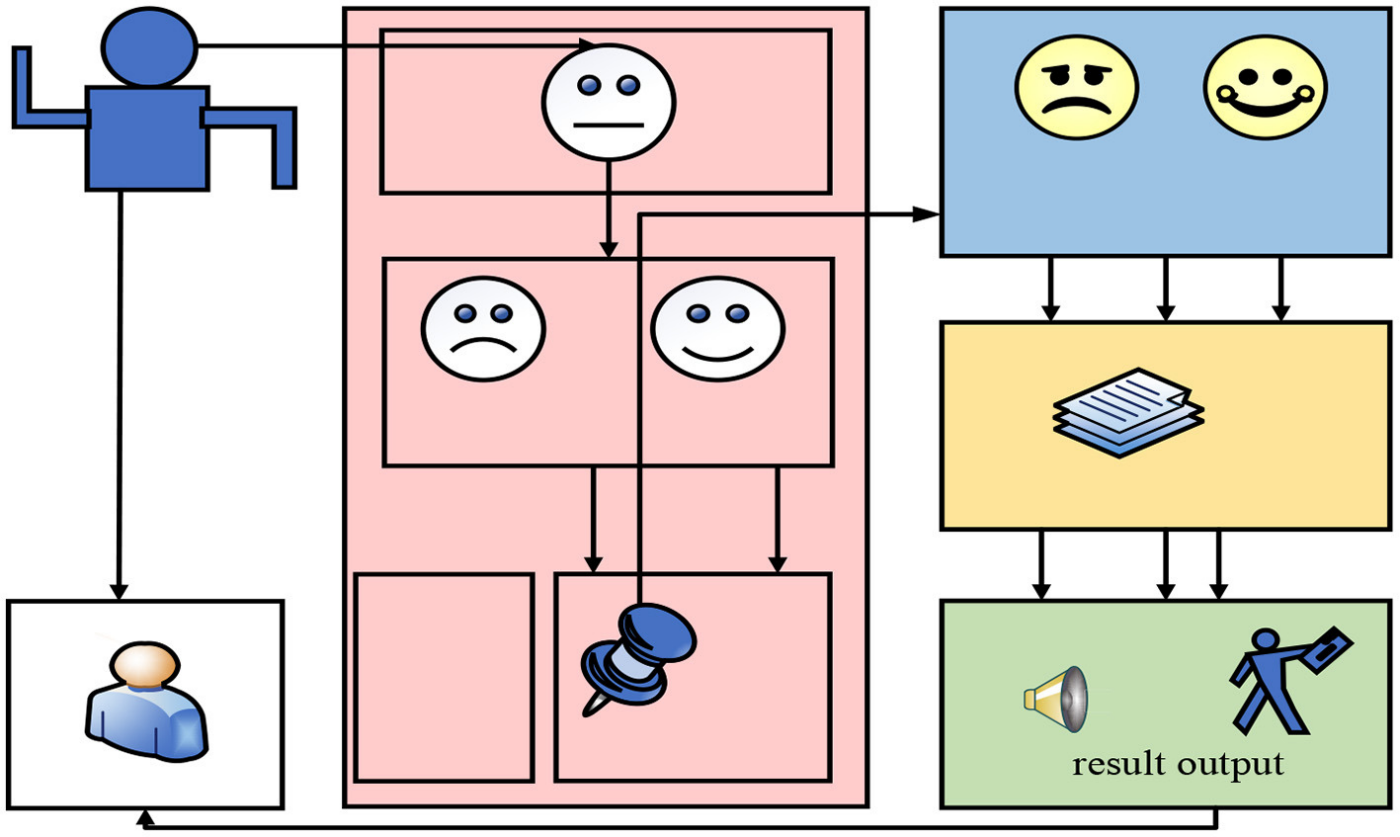
Overall frame diagram of human-computer intelligent interaction system.

In the design of the interaction system between service robots and humans, if we want to give robots and humans to interact emotionally, it is a key link for robots to perceive and understand human emotional expressions (Yu and Wang, 2017). Human emotion expression mainly includes expressions, language and body movements. In this paper, the recognition of facial expressions is mainly used to allow robots to understand human emotion expressions and give corresponding interactive feedback (Kim et al., 2021). The overall design block diagram of the human-computer intelligent interaction system is shown in Figure 2.

As can be seen from Figure 2, the intelligent interaction system based on facial expression recognition is mainly composed of a machine module, an interactive object module, a facial expression recognition module, and a voice and action interaction module. When the interactive object produces various expressions in the self-learning class, the machine will take a screenshot and save it in time for the facial expression recognition module to detect and extract features. Then in the facial expression recognition module, the module will locate and track the key feature points of the face after detecting the face. In this way, effective features can be extracted to represent facial expression changes. While positioning and tracking, the module will send the facial expression features captured from the face to the built-in classifier for cause classification. The reasons are mainly divided into two categories, one is voice interaction, and the other is action interaction. After understanding the classification of students' expressions, secondary emotion recognition will be performed by calling the local database. This secondary recognition process can enhance the discrimination of students' facial expressions. It can improve the facial expression recognition rate to a certain extent, so as to keep in the transformation and development of teachers' teaching mode and improve students' autonomous learning ability.

### Schematic diagram of the field of view of the infrared camera

**Figure 3:**
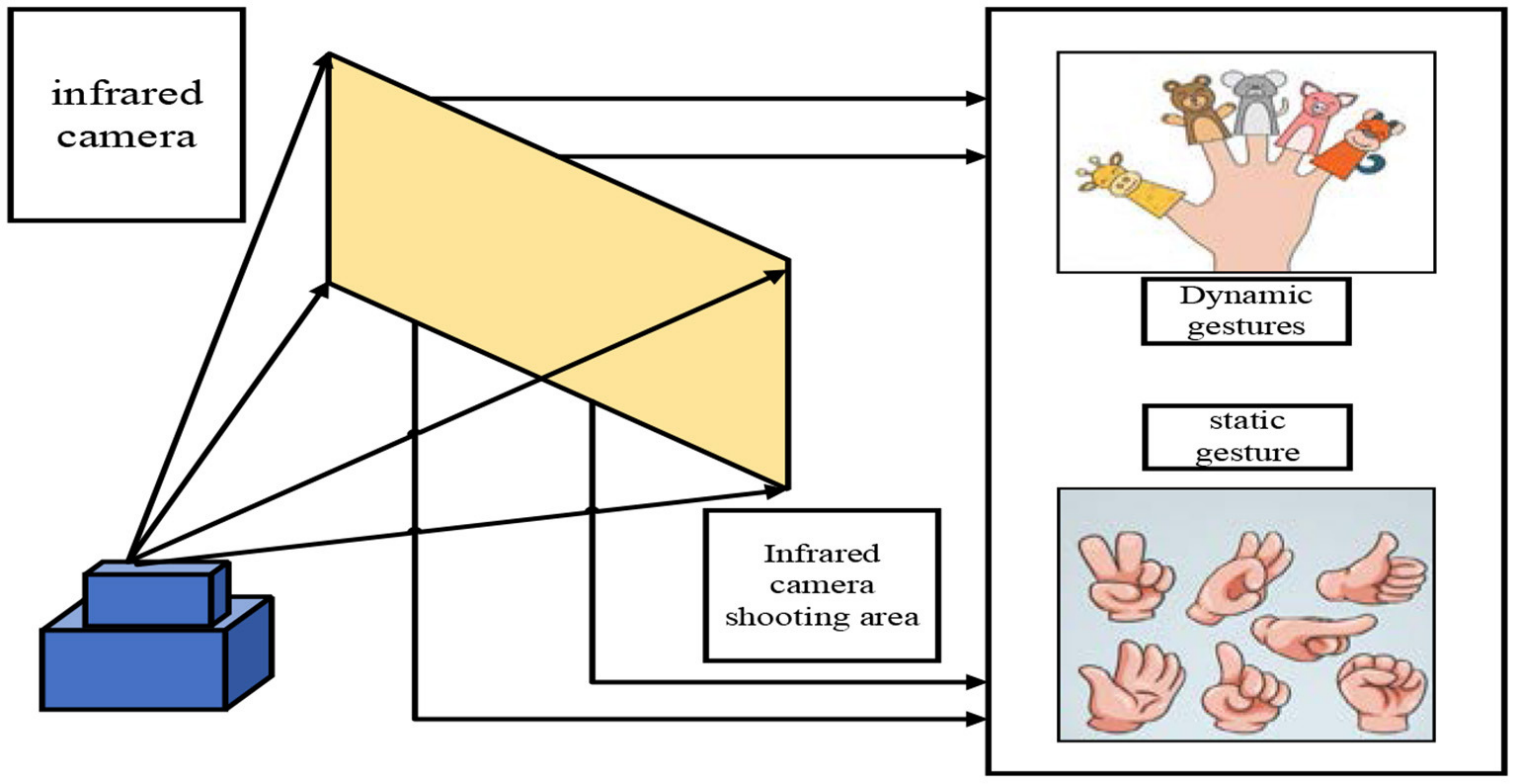
Schematic diagram of the field of view of the infrared camera.

Typically, an infrared sensor acquires a depth map of the scene by emitting energy into the scene and then receiving the scene's reflection of the emitted energy (Shimonishi and Kato, 2017). At present, a relatively mature infrared sensor can obtain scene depth data. It is equipped with a depth camera, including an infrared emission device and an infrared camera, which can directly obtain depth image data with very high resolution. In order to construct the history teaching mode of autonomous learning classroom based on human-computer interaction emotion recognition, this paper uses a deep data action recognition method based on infrared sensors. The specific camera field of view is shown in Figure 3.

As can be seen from Figure 3, objects farther from the sensor have a larger cross-sectional area of the field of view than objects that are closer. This means that the height and width of the image do not correspond one-to-one with the physical location of the sensor's field of view, but the depth value of each pixel corresponds to the distance of objects in the field of view from the sensor. In the autonomous learning classroom under the history teaching mode, students cannot be static all the time. Because students are changing all the time, especially in the process of continuous learning, it is necessary to identify the static and dynamic movements of students. For the expression of a static posture and gesture action, it roughly includes the following elements: the expression of static posture is related to the combination of the spatial position of the skeleton joint points. The understanding of gesture actions is expressed based on the motion trajectories of one or more joint points. The expression of action has the property of time duration, which means that the recognition of student action needs to be based on the duration of a certain time. An action may be expressed by a combination of several sub-actions. For gesture recognition, in addition to tracking the trajectory of some joint points in space and measuring the linear velocity, it may also be necessary to calculate the angular velocity of the elbow joint movement. Using gestures as the means of interaction has the characteristics of being natural, concise and rich. To a certain extent, the students' body movements also express the students' various thoughts and emotions in the study of history. For this reason, students' hand movements should also be constantly paid attention to when constructing the history teaching mode in the autonomous learning classroom.

### Situation-based emotion recognition system

**Figure 4:**
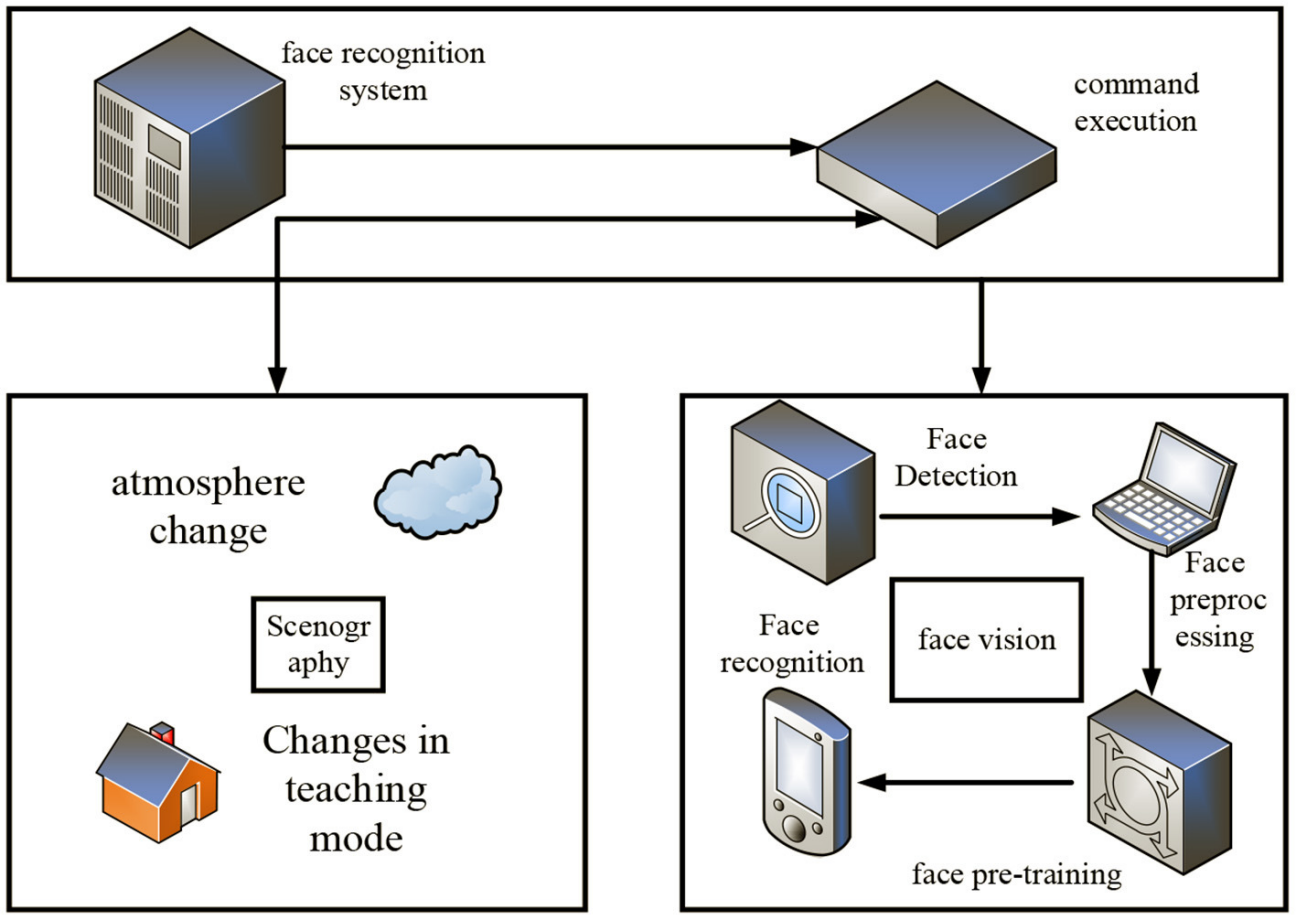
Module diagram of emotion recognition system based on contextualization.

While constructing the history teaching mode of the autonomous learning classroom, we cannot only analyze the emotions of the learning subject (Zhang et al., 2018). It also conducts research on classroom situations of autonomous learning, so as to understand students' emotions at all times. By changing the teaching plan in time, it creates a good learning atmosphere. In order to improve students' autonomous learning ability, this paper analyzes the different needs in the construction of history teaching mode of autonomous learning classroom based on human-computer interaction emotion recognition. This paper studies the emotion recognition system based on context, and its specific modules are shown in Figure 4.

As can be seen from Figure 4, the emotion recognition system based on contextualization is mainly divided into two parts: face vision and sceneization. Face vision includes four modules: face detection, face preprocessing, face training, and face recognition. Scenography includes two modules: learning atmosphere and teaching mode. The face detection module is the basic module of the whole system. Its function is to find faces in pictures with complex backgrounds and obtain the positions of faces. The face preprocessing module further processes the results obtained by face detection. The purpose is to obtain a normalized face image, which is convenient for subsequent feature extraction operations. The core function of the face training module is to perform feature extraction on normalized images, and the extracted face features will be stored as feature files in the form of text files. The core function of face recognition is to compare the extracted features with the existing feature files to determine whether the currently obtained facial emotions exist in the feature files. The atmosphere module and the teaching mode module are mainly changed according to the feature extraction information of the visual part of the face. The emotion recognition system based on situationalization can help history lecturers to a large extent to recognize various emotions of students and change their teaching mode in time.

### The implementation process of the self-learning classroom history teaching mode

**Figure 5:**
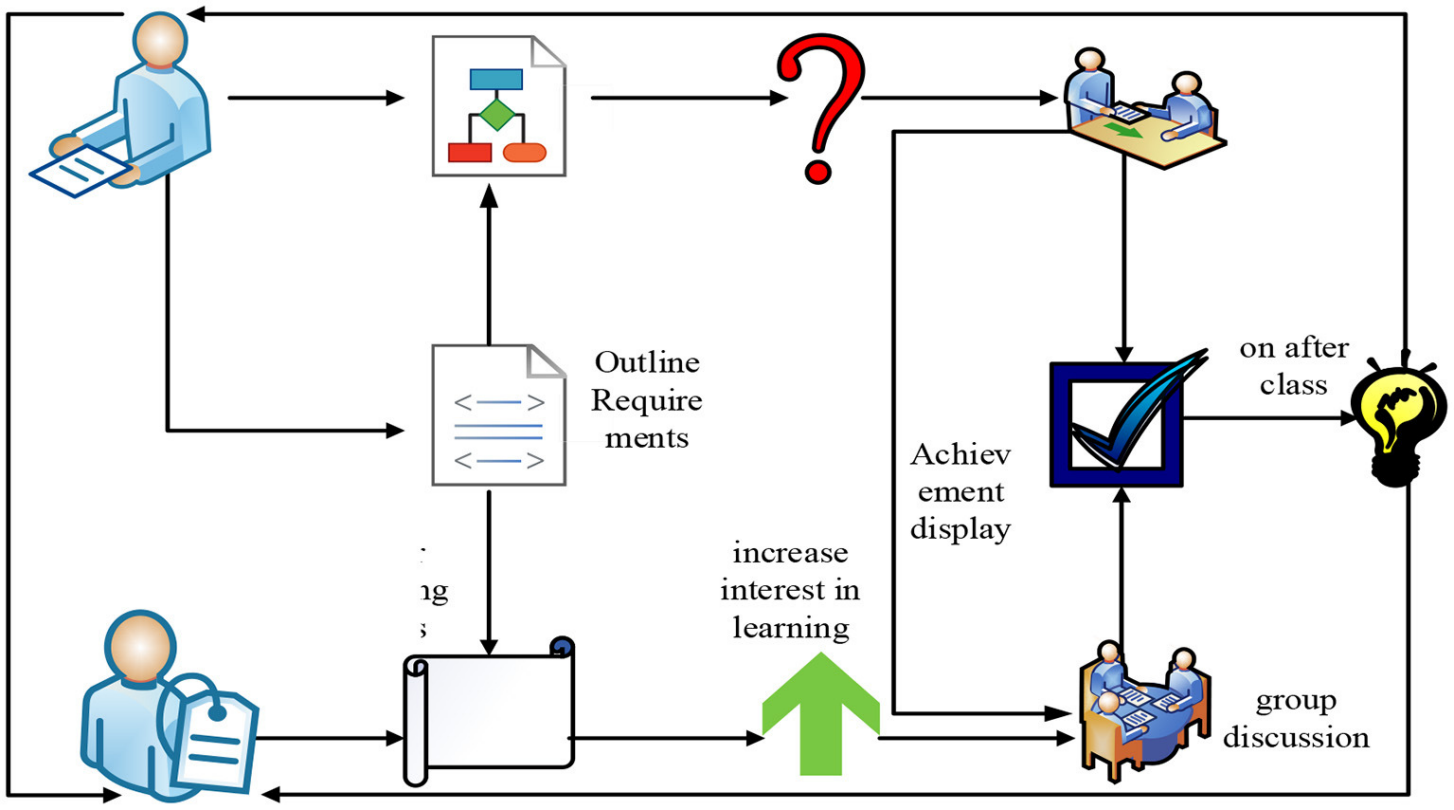
Self-learning classroom history called mode implementation process.

In order to study the construction of the teaching mode of autonomous learning classroom history based on human-computer interaction emotion recognition (Jiang, 2017), it is necessary to conduct in-depth research on the implementation process of the autonomous learning classroom history mode. In order to better construct the autonomous learning classroom under the history teaching mode, this paper studies its implementation process, and the specific implementation process is shown in Figure 5.

As can be seen from Figure 5, the implementation process of the self-learning classroom history teaching mode mainly includes two main subjects: teacher activities and student activities. In the process of implementing the history teaching mode in the autonomous learning classroom, teachers need to conduct a detailed analysis of the syllabus requirements and teaching objectives of history teaching. And it conveys the clear historical learning objectives to the students through the specific syllabus to help students learn. When teachers teach history in autonomous learning classrooms, they need to create learning situations according to specific history teaching models, and put forward and bring in corresponding historical questions. The students should think accordingly and answer the questions raised by the teacher according to the questions raised by the teacher. In this interactive process between teachers and students, students' interest in history learning will be continuously improved. In the history teaching mode of autonomous learning into the classroom, students' learning method is mainly based on group cooperation and exploration. Only when students encounter problems that cannot be solved by themselves, teachers will give timely help. This kind of history teaching mode can stimulate students' learning potential to a great extent and improve the efficiency of teachers' teaching.

## Construction algorithm of the history teaching mode of self-learning classroom

(1) Joint speed


(1) $$\theta =J\begin{array}{c} \;\\ 1 \end{array}\;(p+k\begin{array}{c} \;\\ p \end{array}e\begin{array}{c} \;\\ q \end{array})+\beta v$$

β and *k*_p_ are scalar coefficients; $e\begin{array}{c} q \end{array}$ is the difference between the expected trajectory and the actual trajectory at the end; v is the gradient of the obstacle avoidance potential function.

(2) Joint distance

There is a distance between joints and between joints and obstacles (Li et al., 2017). The calculation formula is:


(2) $$D=\sqrt{(x\begin{array}{c} \;\\ i \end{array}-x\begin{array}{c} \;\\ j \end{array})\begin{array}{c} 2\\ \; \end{array}+(y\begin{array}{c} \;\\ i \end{array}-y\begin{array}{c} \;\\ j \end{array})\begin{array}{c} 2\\ \; \end{array}+(z\begin{array}{c} \;\\ i \end{array}-z\begin{array}{c} \;\\ j \end{array})\begin{array}{c} 2\\ \; \end{array}}$$

Among them, i represents the joint point, j represents the obstacle, and x, y, and z represent the joint distance at different positions.

(3) Obstacle avoidance potential function


(3) $$H(\theta )=\alpha \begin{array}{c} \;\\ m \end{array}\sum_{i=1}^{n+1}\sum_{j=1}^{K}F\begin{array}{c} \;\\ mij \end{array}(\theta )$$

(4) Same-sex charge repulsion


(4) $$F=\frac{q\begin{array}{c} \;\\ 1 \end{array}q\begin{array}{c} \;\\ 2 \end{array}r}{4\pi \varsigma \begin{array}{c} \;\\ 0 \end{array}|r|\begin{array}{c} 3\\ \; \end{array}}$$

Among them, $q\begin{array}{c} 1 \end{array}$ and $q\begin{array}{c} 2 \end{array}$ are the electric quantities of the two charges, and r is the distance between the two charges. According to the formula, the repulsive force between two charges is proportional to the product of their electric charge and inversely proportional to the distance between them.

(5) Repulsion coefficient


(5) $$k(d)=\{\begin{array}{ll} \; & 0\begin{array}{ll} \; & d>D \end{array}\\ \; & \frac{k\begin{array}{l} \;\\ 0 \end{array}}{D}\begin{array}{ll} \; & 0<d<D \end{array} \end{array}$$

Among them, $k\begin{array}{c} 0 \end{array}$is a constant value, which can be determined through multiple experiments.

(6) Gradient of obstacle avoidance potential function


(6) $$v=\partial \sum_{i=1}^{n}\sum_{j=1}^{K}f\begin{array}{c} \;\\ ij \end{array}(\text{χ})$$

Among them, ∂ is a constant value, which is determined by the actual situation.

(7) Angular acceleration


(7) $$\theta \begin{array}{c} \;\\ a \end{array}(t)=\theta \begin{array}{c} \;\\ a0 \end{array}(t)+\beta (I-J)v\begin{array}{c} \;\\ a \end{array}$$

(8) Trajectory optimization objective function

The trajectory optimization objective function has the shortest running time (Peng et al., 2020), and its calculation formula is:


(8) $$\min T=\sum_{a=1}^{n}h\begin{array}{c} \;\\ a \end{array}$$

(9) Fitness function

The design of the fitness function adopts the weighted summation method of each evaluation function, and its calculation formula is:


(9) $$F(h)=\sum_{a=1}^{n}h\begin{array}{c} \;\\ a \end{array}+\zeta \begin{array}{c} \;\\ i \end{array}f\begin{array}{c} i\\ \; \end{array}$$

Among them, $\zeta \begin{array}{c} i \end{array}$ is the undetermined coefficient, which needs to be determined according to the specific situation.

(10) Variant individuals


(10) $$X=X+r(X\begin{array}{c} u\\ \; \end{array}-X)*(1-g/G)\begin{array}{c} c\\ \; \end{array}$$

(11) Color saturation

Colors of various hues and saturations can be obtained by changing the respective intensity ratios of the three primary colors. The calculation formula is:


(11) $$\begin{array}{rcl} C & = & rR+gG+bB \end{array}$$

(12) $$\begin{array}{rcl} r+g+b & = & 1 \end{array}$$

Among them, C represents a certain color, R, G, B represent the three primary colors, and r, g, and b represent the proportional coefficients of each primary color.

(12) Normalized RGB color space

The normalized RGB color space represents the proportional relationship of the three primary colors in the color, and its calculation formula is:


(13) $$\begin{array}{rcl} r & = & \frac{R}{R+G+B} \end{array}$$

(14) $$\begin{array}{rcl} g & = & \frac{G}{R+G+B} \end{array}$$

(15) $$\begin{array}{rcl} b & = & \frac{B}{R+G+B} \end{array}$$

(13) Color space conversion


(16) $$\begin{array}{rcl} H & = & \arccos\left(\frac{\left(R-G\right)+\left(R-B\right)}{2\sqrt{\left(R-G\right)\begin{array}{c} 2 \end{array}+\left(R-B\right)\left(G-B\right)}}\right) \end{array}$$

(17) $$\begin{array}{rcl} H & = & 2\pi -\arccos\left(\frac{\left(R-G\right)+\left(R-B\right)}{2\sqrt{\left(R-G\right)\begin{array}{c} 2 \end{array}+\left(R-B\right)\left(G-B\right)}}\right) \end{array}$$

(14) Information entropy


(18) $$E=-\sum_{j=l}^{L}p\begin{array}{c} \;\\ j \end{array}\ln p\begin{array}{c} \;\\ j \end{array}$$

(15) Normalized histogram


(19) $$HI=\sum_{j=1}^{L}\min(H\begin{array}{c} \;\\ S \end{array}(j),H\begin{array}{c} \;\\ N \end{array}\;(j))$$

(20) $$HCE=\sum_{j=1}^{L}\frac{(H\begin{array}{c} \;\\ S \end{array}(j)-H\begin{array}{c} \;\\ N \end{array}(j))\begin{array}{c} 2\\ \; \end{array}}{H\begin{array}{c} \;\\ S \end{array}(j)-H\begin{array}{c} \;\\ N \end{array}(j)}$$

$H\begin{array}{c} S \end{array}$and $H\begin{array}{c} N \end{array}$ represent the normalized histograms of skin and non-skin, respectively.

## Construction method of the history teaching mode in the autonomous learning classroom

### Literature research method

The literature research method mainly refers to the method of collecting, identifying and arranging literature, and forming a scientific understanding of the facts through the study of literature. At the same time, the method of literature research is also an ancient and vigorous scientific research method. By going to the library to look up books, journals and newspapers about self-learning classrooms, history teaching models and human-computer interaction emotion recognition, this paper browses the relevant teaching websites that build history teaching models. It collects, organizes and reads and analyzes the relevant materials and policies and regulations for the construction of the history teaching mode of the autonomous learning classroom. By sorting and classifying the collected data, it lays a theoretical foundation for writing this article.

### Experimental analysis method

In order to obtain effective data information, this paper conducts investigation, research and experiment on the class of a middle school. This paper selects students from classes 1–8 from the middle school to experience different history teaching modes, and evaluates these teaching modes after the end. At the same time, participate in the quiz after experiencing different teaching modes to obtain specific experimental data. The number of students in classes 1–8 is the same. The data in this paper is mainly based on the evaluation of different teaching modes by the middle school students, combined with the online survey data. In order to make the research more convenient, this paper names the different history teaching modes, respectively. It is mainly named Mode 1, Mode 2, Mode 3, Mode 4, and Mode 5. Among them, mode 5 is the history teaching mode of autonomous learning classroom constructed based on human-computer interaction emotion recognition.

### Data analysis method

In order to facilitate the specific results and the construction and development of the history teaching model, this paper will use the 100% system to analyze the collected data specifically, so as to facilitate the construction and research of the history teaching model in the autonomous learning classroom.

## Experimental on the teaching mode of classroom history in autonomous learning

### Students' evaluation of different teaching modes

**Figure 6:**
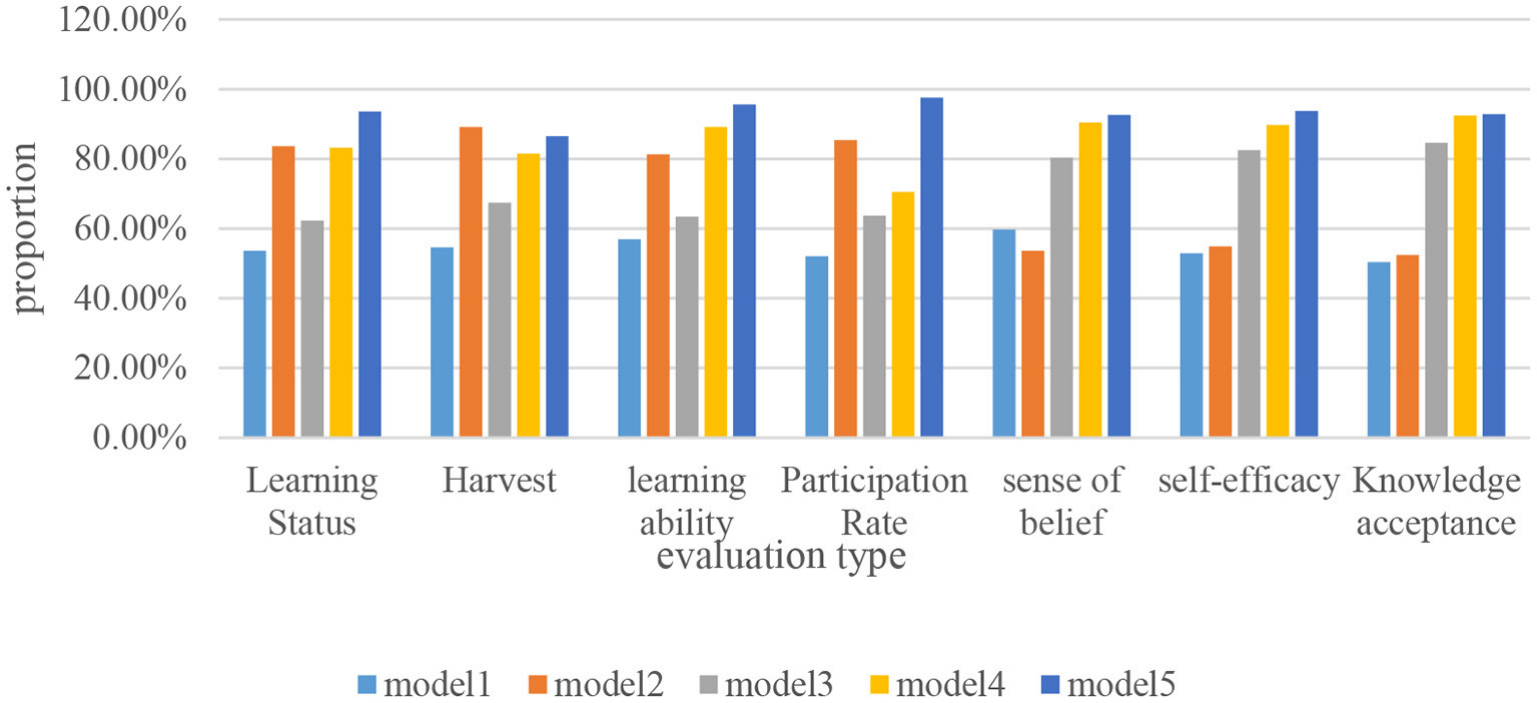
Students' evaluation of different teaching modes.

By improving students' autonomous learning ability and creating a good learning environment, it is the main purpose of constructing the history teaching mode of autonomous learning classroom. In order to make the construction of self-learning classroom history teaching mode more meaningful, based on the evaluation of the overall learning under different history teaching modes by the subject of learning, this paper studies the construction of a history teaching mode for autonomous learning in classrooms based on human-computer interaction emotion recognition. The specific evaluation data is shown in Figure 6.

It can be seen from Figure 6 that students mainly evaluate different history teaching modes in seven aspects: learning status, gain, learning ability, participation rate, belief, self-efficacy, and knowledge acceptance. In terms of learning status, the proportion of mode 1 is the lowest, only about 50%, far less than the proportion of other teaching modes. In terms of harvest, the proportion of Mode 2 is more than 80%, which is much higher than that of other teaching modes. It shows that students can gain a lot from the teaching of Mode 2. In terms of learning ability, Mode 1 and Mode 3 have the lowest proportions, which shows that students cannot improve their autonomous learning ability well in these two teaching modes. In terms of participation rate, Mode 2 and Mode 5 have the two highest proportions of all teaching modes. It shows that students feel that they can get a strong sense of participation in these two teaching modes. In terms of belief, self-efficacy and knowledge acceptance, Mode 3, Mode 4 and Mode 5 all have the highest proportions. Among them, the proportion of mode 4 is much higher than that of mode 1, mode 2 and mode 3. However, it is still inferior to Mode 5, indicating that students have a stronger sense of learning belief in Mode 4. Overall, students rated History Teaching Mode 5 as the highest among the five teaching modes, and they agreed that Mode 5 was the best teaching mode. However, the evaluation of Mode 5 in terms of harvesting degree is relatively low, and it still needs continuous improvement.

### Comparative analysis between different history teaching modes

**Figure 7:**
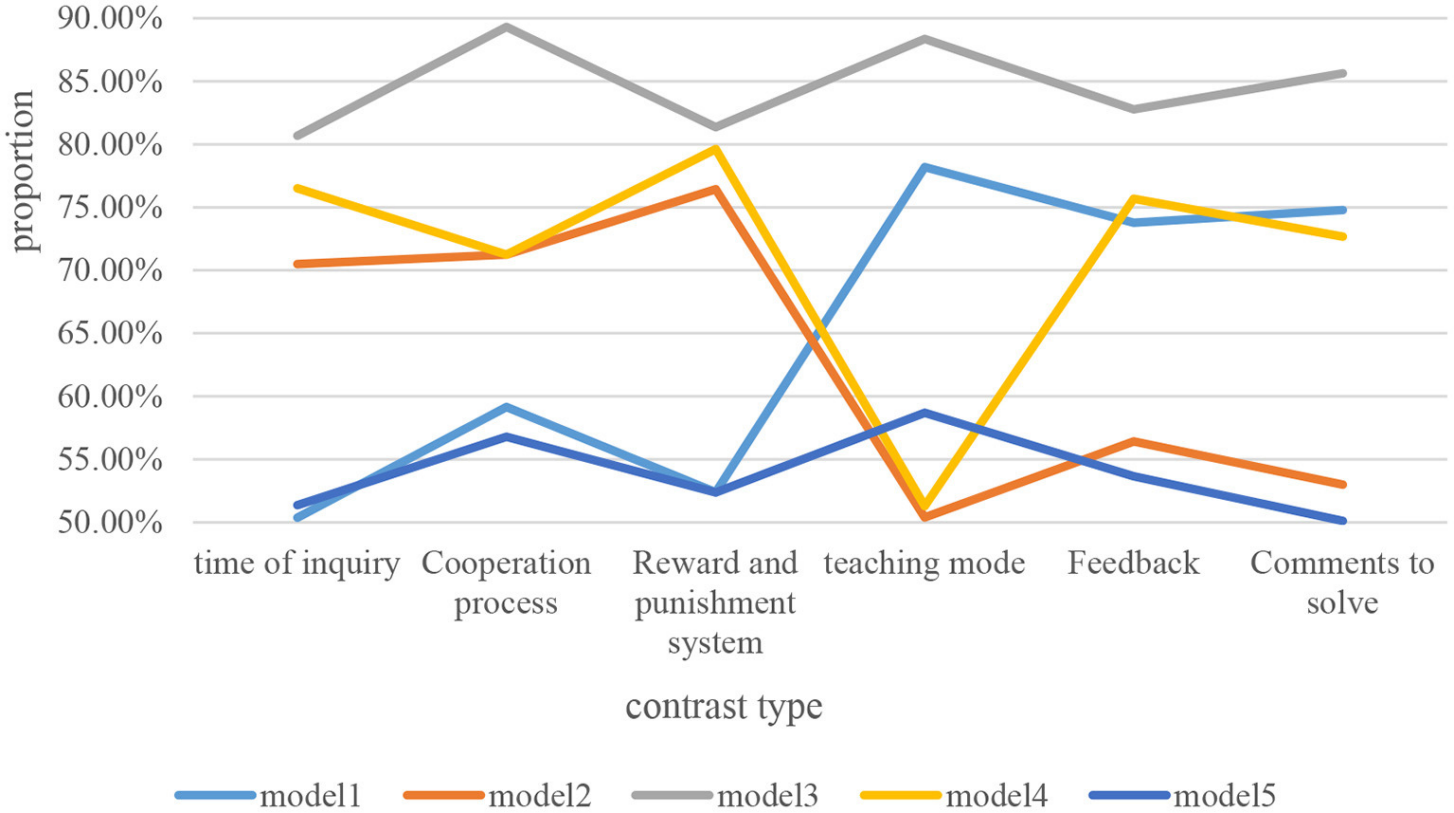
Comparative analysis between different history teaching modes.

With the continuation of the epidemic and the continuous development and changes of the times, the history teaching mode of independent learning classrooms has become the focus of social attention. How to impart unlimited historical knowledge in limited autonomous learning classrooms is an urgent problem to be solved in the current social development. In order to better build a history teaching model for autonomous learning classrooms, this paper compares and analyzes different history teaching models, and the specific data are shown in Figure 7.

As can be seen from Figure 7, this paper mainly compares and analyzes different history teaching modes from six aspects: exploration time, cooperation process, reward and punishment system, comprehensive teaching, feedback and problem solving. In terms of exploration time and reward and punishment system, Mode 1 and Mode 5 have the lowest proportions because their proportions are only between 50 and 60%. This shows that students need to spend more time in the two history teaching modes to understand the historical knowledge taught by teachers. And there is no better reward and punishment system to encourage students to learn, which easily causes students to be disgusted with learning history. In terms of the cooperation process, the proportion of Mode 3 is the highest, as high as about 90%, indicating that students are more likely to cooperate and learn with other students in the teaching mode of Mode 3. In terms of teaching integrity, the proportion of Mode 2 and Mode 4 is about 50%, which is the lowest among all teaching modes. It means that if teachers teach knowledge in these two teaching modes, it is easy to cause students to accept incomplete historical knowledge. In terms of feedback and problem solving, Mode 2 and Mode 5 have the lowest proportions compared to other teaching modes, indicating that teachers and students are prone to lack of interaction and knowledge bias in these two modes. Overall, Mode 1 is the best, and Mode 5 is the worst of all modes. It needs continuous improvement and development to enhance the interaction between teachers and students.

### Comparative analysis of the effectiveness of different teaching modes

**Figure 8:**
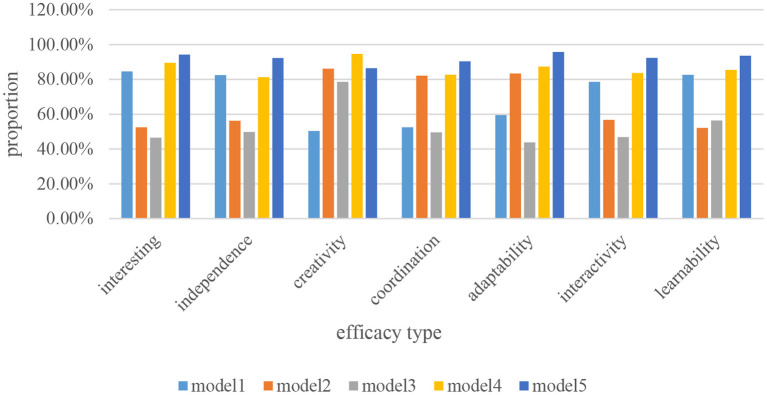
Comparative analysis of effectiveness between different teaching modes.

There are many different types of history teaching modes at present, and different types of history teaching modes have their different efficacy and characteristics. In order to better construct the history teaching mode of autonomous learning classroom, this paper compares and analyzes the effectiveness of different teaching modes, and the specific analysis data is shown in Figure 8.

This paper mainly compares and analyzes different history teaching modes from seven aspects: interest, independence, creativity, coordination, adaptability, interaction and learning. It can be seen from Figure 8 that in terms of interest, the proportions of Mode 2 and Mode 3 are the lowest. It shows that students may be difficult to obtain higher interest in these two teaching modes, so that they can easily lose their interest in learning history. In terms of independence, the proportion of Mode 1, Mode 3 and Mode 4 is about 20% higher than that of Mode 2. It shows that the independence of these three modes is relatively high, which can greatly improve the teaching efficiency. In terms of creativity, coordination and adaptability, the proportions of mode 1 and mode 3 are far inferior to the other three teaching modes, and the effectiveness in these aspects is the lowest. In terms of interactivity, the proportions of Mode 1, Mode 3 and Mode 5 are relatively high compared to the other two teaching modes, indicating that students can get close interaction with history teachers in these three modes. In terms of learning, the proportions of Mode 2 and Mode 3 are the lowest among all history teaching modes, indicating that it is difficult for students to learn more historical knowledge in these two teaching modes. On the whole, the proportions of Mode 4 and Mode 5 are the two teaching modes with relatively high proportions and stable in all aspects among all historical teaching modes, which are more suitable for teachers and students to experience personally.

### The recognition rate of different emotions in different teaching modes

**Figure 9:**
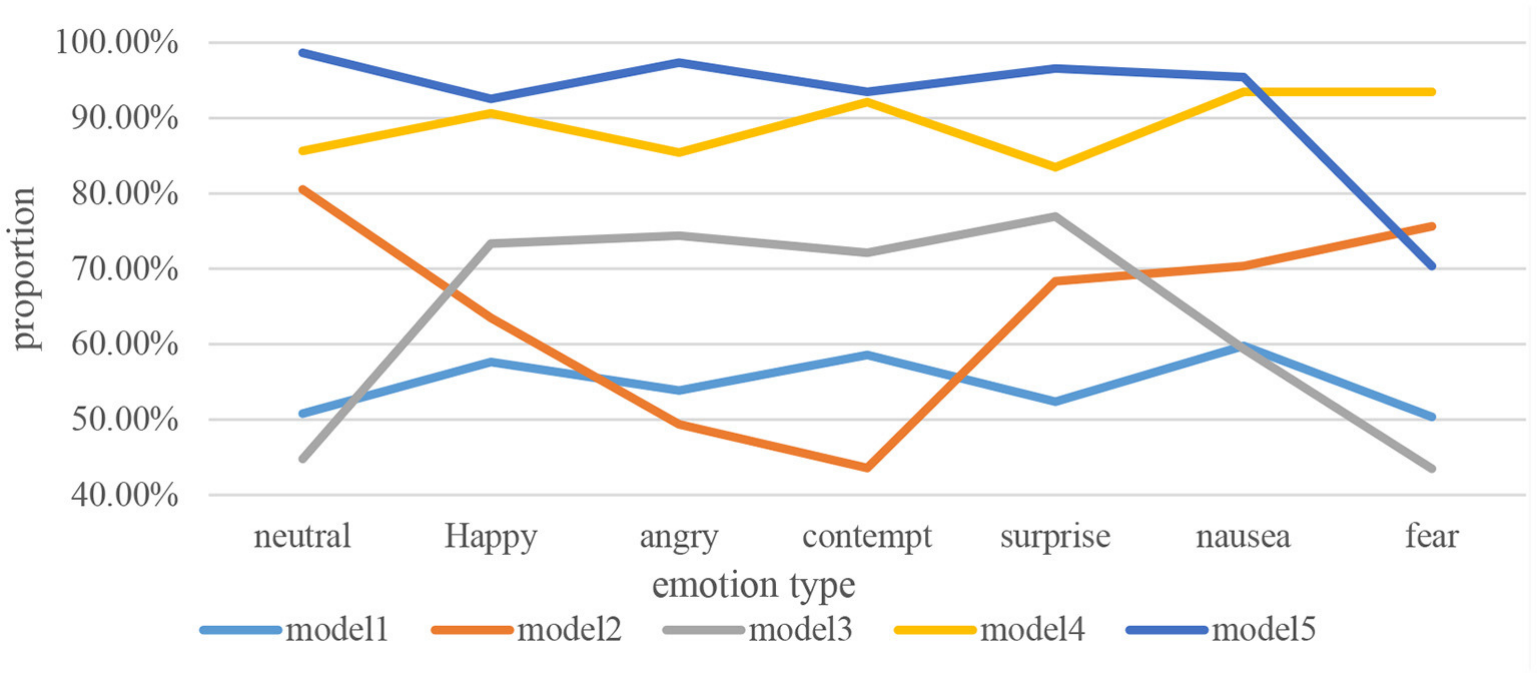
Recognition rates of different emotions for different teaching modes.

Identifying different emotions is the biggest support and theoretical basis for improving and constructing the history teaching mode of different autonomous learning classrooms. In order to better construct the history teaching mode of autonomous learning classroom based on human-computer interaction emotion recognition, this paper conducts in-depth research on the recognition rate of different emotions in different teaching modes. It is in order to provide teachers with a good reference of history teaching mode, and it constantly improves its own teaching mode. Its specific data is shown in Figure 9.

As can be seen from Figure 9, students' emotions are roughly divided into seven types: neutral, happy, angry, contemptuous, surprised, disgusted, and fearful. In the recognition of neutral emotions, the recognition rates of Mode 1 and Mode 3 are the lowest, indicating that these two modes are difficult to respond to neutral emotions. In the recognition of happy and angry emotions, the recognition rate of Mode 4 and Mode 5 is over 85%, which is the highest recognition rate of happy and angry emotions among all historical teaching modes. In terms of the recognition of contemptuous emotion, the recognition rates of Mode 1 and Mode 2 are the lowest compared with other teaching modes, indicating that these two teaching modes cannot accurately identify the students' emotions of contempt. In the recognition of surprise emotion, the recognition rates of Mode 3, Mode 4 and Mode 5 are relatively high among all the history teaching modes. It shows that these three modes can be well-applied to the recognition of surprise emotion. In terms of the recognition of nausea, Mode 2 has a higher recognition rate than Mode 1 and Mode 3, about 70%. In the recognition of fear emotion, the recognition rate of mode 5 is far lower than that of mode 2 and mode 4. It shows that if people want to recognize fear, it is best to reduce the use of the history teaching mode of Mode 5. But overall, Mode 5 has the highest accuracy in other emotion recognition among all teaching modes. To a large extent, it meets the needs of current human-computer interaction emotion recognition.

### Score rate of student history under different teaching modes

**Figure 10:**
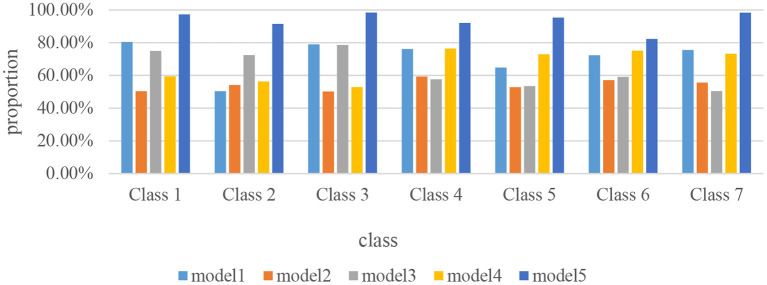
Score rate of student history under different teaching modes.

The main purpose of teaching history to students by using different teaching modes in self-learning classrooms is to enable students to receive a good history education to the greatest extent. And the best way to test whether students are getting a good history education is after they have experienced different teaching modes. By giving these students a quiz in the classroom, their specific score data was compiled for easy analysis. Its specific score data is shown in Figure 10.

As can be seen from Figure 10, this paper conducted a classroom quiz for students in Class 1, Class 2, Class 3, Class 4, Class 5, Class 6 and Class 7. On the whole, the students in Class 1 have the lowest scoring rate in Mode 2 and Mode 3, indicating that these two teaching modes are not suitable for the use of Class 1. The score rate of Class 2 under the teaching modes of Mode 1, Mode 2, and Mode 4 is relatively low, indicating that the history teaching of Class 2 is not suitable for using these three modes. The equivalence rate of Class 3 in the teaching modes of Mode 1, Mode 3, and Mode 5 is relatively high, mostly around 80% or above. It shows that compared with other modes, these three modes are the most suitable for the history teaching of Class 3. Class 4 and 5 have the highest scoring rates in Mode 4 and Mode 5 compared to the other three teaching modes. It shows that the history teaching of Class 4 and Class 5 is the most suitable for the two teaching modes of Mode 4 and Mode 5. Class 6 and Class 7 have the lowest scoring rate in Mode 3, Mode 4, and Mode 5, while the scoring rate in Mode 1 and Mode 5 is relatively high. It shows that Class 6 and Class 7 are most suitable for the history teaching mode of Mode 1 and Mode 5. On the whole, Mode 5 is a history teaching mode that is more applicable in all classes. Because the students in each class generally scored more than 85% in the teaching mode of Mode 56, which is the highest among all modes.

## Experimental results of the self-learning classroom history teaching mode

The history teaching mode of self-learning classroom is a new teaching mode that has emerged in recent years. It can greatly reduce teachers' teaching pressure and improve teaching efficiency, thereby improving students' interest in learning and improving students' learning efficiency. However, the current history teaching mode of autonomous learning classroom has great defects. It is because it cannot feel the emotional changes of students' learning to a great extent. In order to solve this problem, this paper constructs the history teaching mode of autonomous learning classroom based on the emotion recognition technology of human-computer interaction.

(1) This paper studies the evaluations made by students for different teaching modes. The main purpose of constructing the history teaching mode of autonomous learning classroom in this paper is to provide students with a good history learning environment. The results of the research on student evaluations show that the students' learning status under the teaching mode of Mode 1 is relatively poor, and they cannot concentrate on learning historical knowledge. Students have a relatively high degree of harvest in the history teaching mode under Mode 2, and can gain a lot of historical knowledge and other things. Students' learning ability under the teaching mode of Mode 3 is the lowest, and it is difficult to improve their autonomous learning ability to a large extent. Students' belief, self-efficacy and knowledge acceptance of the history teaching modes under Mode 3, Mode 4 and Mode 5 are relatively high, and Mode 5 is a history teaching mode that students generally evaluate highly.

(2) This paper compares and analyzes different history teaching modes. The research results show that students need to spend more time in mode 1 and mode 2 to understand the historical knowledge taught by teachers, and the learning efficiency in these two modes is relatively poor. Students were more likely to cooperate in mode 3 than in the other modes. It shows that if students study in the teaching mode of Mode 2 and Mode 4, it is easy to accept the phenomenon of incomplete historical knowledge. The feedback and problem solving of teachers and students in Mode 2 and Mode 5 are relatively poor. Overall, Mode 5 is the worst performing of all modes and needs continuous improvement and development.

(3) This paper compares and analyzes the effectiveness of different teaching modes. The results of the study showed that Mode 2 and Mode 3 were the least interesting. The independence of mode 1, mode 3 and mode 4 is about 20% higher than that of mode 2, indicating that the independence of these three modes is relatively high. Mode 1 and Mode 3 have relatively low proportions in terms of creativity, coordination and adaptability, which cannot fully cultivate students' autonomy. The interactivity of Mode 5 is the highest among all teaching modes, and students can get close interaction with history teachers in this mode. On the whole, Mode 5 is a history teaching mode that is more suitable for teachers and students to experience personally among all the history teaching modes.

(4) Research on the recognition rate of different teaching modes for different emotions. The results of the study showed that Mode 1 and Mode 3 had the lowest recognition rates for neutral emotions, and it was difficult to respond to them. Mode 4 and Mode 5 have better recognition rates for happy and angry emotions, and can easily identify these two emotions. Mode 1 and Mode 2 have a relatively low recognition rate for contempt, and cannot accurately identify the emotion. On the whole, except for Mode 5, several other history teaching modes have more or less some advantages and disadvantages. Only Mode 5 largely meets the needs of current human-computer interaction emotion recognition.

(5) This paper studies the scoring rate of students' history under different teaching modes. The research results show that, on the whole, the teaching modes of Mode 2 and Mode 3 are not suitable for use in Class 1. The history teaching of class 2 is not suitable for the three modes of teaching mode 1, mode 2 and mode 4. The history teaching mode of Mode 2 and Mode 4 is the least suitable for the history teaching of Class 3 among all modes, but it is suitable for the history teaching of Class 4 and 5. Class 6 and Class 7 are more suitable for the history teaching mode of Mode 1 and Mode 5. On the whole, in addition to Mode 5, several other teaching modes have their own shortcomings.

## Conclusion

With the continuous development of human-computer interaction emotion recognition technology, many people hope to use this technology to build a history teaching mode in autonomous learning classrooms, so as to cultivate students' autonomous learning ability. It reduces the pressure of teaching history teachers. However, due to the current social and economic constraints, the research on human-computer interaction emotion recognition technology is still very limited. It is not to mention that it is applied to the construction of the history teaching mode of the autonomous learning classroom. However, because of the unexpected emergence and continuation of the epidemic, the issue of applying human-computer interaction emotion recognition technology to the construction of a history teaching model for autonomous learning classrooms has been put on the agenda. In order to promote the construction, research and development of the history teaching mode in the autonomous learning classroom, this paper applies the human-computer interaction emotion recognition technology to its construction. In addition, in order to better construct the history teaching mode, this paper also studies the overall framework of the human-computer interaction emotion recognition system and the overall framework of the human-computer intelligent interaction system. At the same time, this paper uses the infrared camera field of view technology and the emotion recognition system based on context to provide technical support for the history teaching model constructed in this paper. At the end of this paper, it also studies the implementation process of the history teaching mode in the autonomous learning classroom. Of course, there are still some shortcomings in this paper, which will be improved and perfected in the future development.

## Data availability statement

The original contributions presented in the study are included in the article/supplementary material, further inquiries can be directed to the corresponding author/s.

## Author contributions

CJ: writing—original draft preparation. SZ: editing data curation and supervision. All authors contributed to the article and approved the submitted version.

## Conflict of interest

The authors declare that the research was conducted in the absence of any commercial or financial relationships that could be construed as a potential conflict of interest.

## Publisher's note

All claims expressed in this article are solely those of the authors and do not necessarily represent those of their affiliated organizations, or those of the publisher, the editors and the reviewers. Any product that may be evaluated in this article, or claim that may be made by its manufacturer, is not guaranteed or endorsed by the publisher.
